# Are Mindful Exercises Safe and Beneficial for Treating Chronic Lower Back Pain? A Systematic Review and Meta-Analysis of Randomized Controlled Trials

**DOI:** 10.3390/jcm8050628

**Published:** 2019-05-08

**Authors:** Liye Zou, Yanjie Zhang, Lin Yang, Paul D. Loprinzi, Albert S. Yeung, Jian Kong, Kevin W Chen, Wook Song, Tao Xiao, Hong Li

**Affiliations:** 1 Lifestyle (Mind-Body Movement) Research Center, College of Sports Science, Shenzhen University, Shenzhen 518060, China; liyezou123@gmail.com (L.Z.); Qigong4us@hotmail.com (K.W.C.); 2 Health and Exercise Science Laboratory, Institute of Sports Science, Seoul National University, Seoul 08826, Korea; elite_zhangyj@163.com (Y.Z.); songw3@snu.ac.kr (W.S.); 3 Cancer Epidemiology and Prevention Research, Alberta Health Services, Calgary, AB T2S 3C3, Canada; lin.yang@ahs.ca; 4 Departments of Oncology and Community Health Sciences, Cumming School of Medicine, University of Calgary, Calgary, AB T2N 4Z6, Canada; 5 Department of Health, Exercise Science and Recreation Management School of Applied Sciences, The University of Mississippi, Oxford, MS 36877, USA; pdloprin@olemiss.edu; 6 Department of Psychiatry, Massachusetts General Hospital, Harvard Medical School, Boston, MA 02114, USA; ayeung@mgh.harvard.edu (A.S.Y.); JKONG2@mgh.harvard.edu (J.K.); 7 Institute on Aging, Seoul National University, Seoul 08826, Korea; 8 College of Mathematics and Statistics, Shenzhen University, Shenzhen 518060, China; 9 Shenzhen Key Laboratory of Affective and Social Cognitive Science, College of Psychology and Sociology, Shenzhen University, Shenzhen 518060, China; 10 Shenzhen Institute of Neuroscience, Shenzhen 518057, China

**Keywords:** Tai Chi, Yoga, Qigong, mind-body therapy, exercise, mind-body medicine, low back pain

## Abstract

Background: Chronic low back pain (CLBP) is a common health issue worldwide. Tai Chi, Qigong, and Yoga, as the most widely practiced mindful exercises, have promising effects for CLBP-specific symptoms. Objective: We therefore conducted a comprehensive review investigating the effects of mindful exercises versus active and/or non-active controls while evaluating the safety and pain-related effects of mindful exercises in adults with CLBP. Methods: We searched five databases (MEDLINE, EMBASE, SCOPUS, Web of Science, and Cochrane Library) from inception to February 2019. Two investigators independently selected 17 eligible randomized controlled trials (RCT) against inclusion and exclusion criteria, followed by data extraction and study quality assessment. Standardized mean difference (SMD) was used to determine the magnitude of mindful exercises versus controls on pain- and disease-specific outcome measures. Results: As compared to control groups, we observed significantly favorable effects of mindful exercises on reducing pain intensity (*SMD* = −0.37, 95% CI −0.5 to −0.23, *p* < 0.001, *I*^2^ = 45.9 %) and disability (*SMD* = −0.39, 95% CI −0.49 to −0.28, *p* < 0.001, *I*^2^ = 0 %). When compared with active control alone, mindful exercises showed significantly reduced pain intensity (*SMD* = −0.40, *p* < 0.001). Furthermore, of the three mindful exercises, Tai Chi has a significantly superior effect on pain management (*SMD*= −0.75, 95% CI −1.05 to −0.46, *p* < 0.001), whereas Yoga-related adverse events were reported in five studies. Conclusion: Findings of our systematic review suggest that mindful exercises (Tai Chi and Qigong) may be beneficial for CLBP symptomatic management. In particular, Tai Chi appears to have a superior effect in reducing pain intensity irrespective of non-control comparison or active control comparison (conventional exercises, core training, and physical therapy programs). Importantly, training in these mindful exercises should be implemented with certified instructors to ensure quality of movement and injury prevention.

## 1. Introduction

Low back pain is a common health issue worldwide, but notably, prevention and treatment of chronic low back pain (CLBP) is a major public health concern [1,2]. It has been widely recognized as the leading cause of disability, affecting work performance and general psychosomatic health and is associated with substantial economic and societal burden [2]. The estimated lifetime prevalence of CLBP is 12% to 33% in industrialized countries (period prevalence: 22% to 65% per year) [3]. The prevalence rate of CLBP is higher in adults than children and adolescents [4], particularly among the working population [5]. CLBP is widely treated with medications (e.g., nonsteroidal anti-inflammatory drug, analgesic, and muscle relaxant) to relieve pain, decrease inflammation, and reduce muscle tension [6]. However, these treatments may increase the likelihood of falls and drug-related side effects (e.g., mood disturbance, nausea, seizure, and/or tachycardia) among patients [6,7]. Furthermore, the long-term use of medications remains financially unaffordable in economically disadvantaged areas [7]. Other non-pharmacological treatments, such as physical therapy [8,9], spinal manipulation [10], and physical activity or exercise [11,12,13], have shown promising effects on improving CLBP-specific symptoms.

Tai Chi, Qigong (e.g., Baduanjin, Yijingjin, and Wuqinxi), and Yoga, also known as mindful exercises, are light-to-moderate intensity physical activities and have recently been popularized in both the fitness industry and clinical setting for disease prevention and symptomatic management [14,15,16,17]. Mindful exercises are typically performed at a slow pace, simultaneously integrated with mental focus on muscle and movement sense, rhythmic abdominal diaphragmatic breathing, and meditation [18,19,20,21]. These modalities may complement or act as an alternative practice to conventional rehabilitation programs [22,23,24]. Mindful exercises are beneficial for symptomatic management in a variety of diseases, such as multiple sclerosis [25,26], autism spectrum disorder [27], balance disorder [28,29], ankylosing spondylitis [30], mental illness [31,32], cerebrovascular disease [33], fibromyalgia [34], and knee osteoarthritis [35].

Recently, research has investigated the effects of mindful exercises in adults with CLBP. With the increasing number of experimental studies on this topic, two reviews were subsequently performed and published in 2013 [36,37]. Notably, these two systematic reviews only included eight to 10 randomized controlled trials (RCT) and focused on Yoga alone. Secondly, meta-analysis was only possible for the Yoga interventions versus non-active controls due to the small number of trials, lacking a direct comparison to active control conditions like conventional exercises or guideline-endorsed treatments. Thirdly, previous reviews simply evaluated the effectiveness of Yoga, but the safety of the broader mindful exercises in adults with CLBP still remains unknown. To fill these knowledge gaps, we therefore conducted an updated systematic review that includes all three most popular mindful exercises versus active and/or non-active controls while evaluating the safety and efficacy of mindful exercises in adults with CLBP.

## 2. Methods

### 2.1. Search Strategy

**Figure 1:**
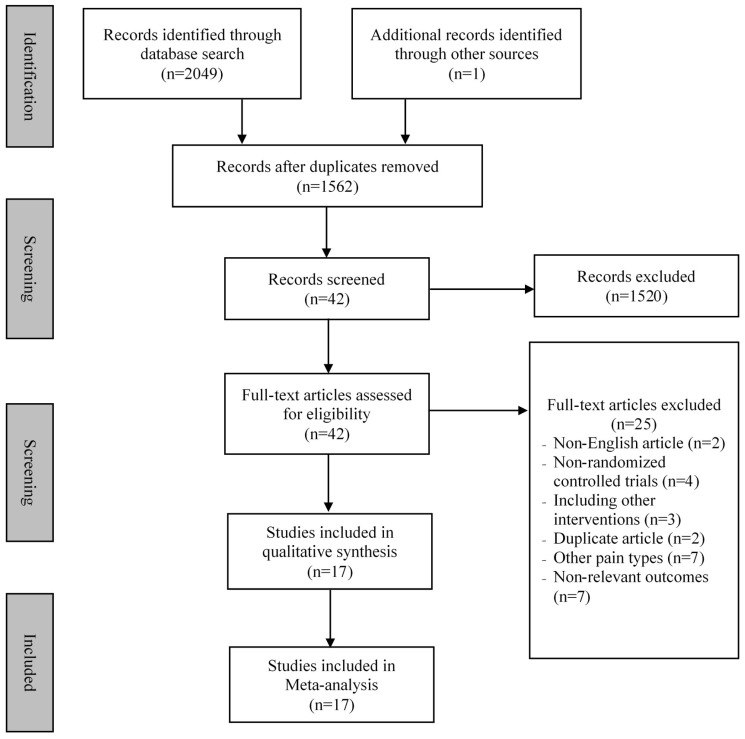
Flow chart of study searching.

Two investigators independently searched five databases (MEDLINE, EMBASE, SCOPUS, Web of Science, and Cochrane library) from the inception to February 2019. We used two groups of keywords: (1) “Tai Chi” OR “Tai Chi Chuan” OR “Taiji” OR “Qigong” Or "Chi Kung“ OR ”Qi Gong“ OR “Baduanjin” OR “Yijinjing” OR “Wuqinxi” OR “Yoga” OR “mind-body”, OR “mindful exercise”; (2) “low back pain” OR “lower back pain” OR “back pain” OR “low back ache”. Hand-searching was performed to identify relevant publications from the reference lists of eligible original articles and reviews. In addition to two separate investigators independently searching the five above-mentioned databases, these investigators also independently screened the titles and abstracts of the potentially eligible articles (described below). Full details on the search strategy and retrieval process are shown in Figure 1.

### 2.2. Inclusion and Exclusion Criteria

In the present review, studies were only considered eligible if they: (1) were RCTs; (2) recruited adults diagnosed with CLBP (low back pain lasting or recurring for longer than 3 months [38]; (3) used at least one type of mindful exercise (e.g., Tai Chi, Qigong, and Yoga) or their combination as an intervention program; (4) included a control group using any form (e.g., aerobic exercise, self-care book, waitlist, or no treatment) other than mindful exercise; (5) reported at least one health outcome associated with disease-specific symptoms like pain, functional ability, or depression. Exclusion criteria were: (1) specific causes (e.g., spinal canal stenosis or herniated disc); (2) mindful exercise integrated with other treatments, like core training; (3) unobtainable data for calculating effect size (ES); (4) other types of publications, such as a case-study, observational study, or review articles.

### 2.3. Data Extraction and Quality Assessment

Detailed information of each included study were independently extracted by the two investigators and a third reviewer was consulted to reach consensus by discussion. Extracted information included the first author and year of publication, characteristics of participants (sample size and mean age), intervention protocol (mindful exercise, control type, and intervention duration), outcome measure (pain, disability, and/or depression), and reporting of an adverse event. In addition to descriptive information, the same investigators extracted the quantitative data for ES calculation.

Two investigators independently assessed methodological quality using the Physiotherapy Evidence Database (PEDro) scale. This scale consists of 11 items, including eligibility criteria, random allocation, allocation concealment, baseline equivalence, blinded assessor(s), blinded participants, blinded instructor, retention rate of ≥85%, intention-to-treat analysis (ITT), between group statistical comparisons, and point estimates of at least one set of outcome measures. One point is awarded for meeting each evaluation requirement. Since this review included all adults diagnosed with CLBP, the first eligibility criteria was not considered. Thus, each study could reach a maximum of 10 points: excellent (9–10 points), good (6–8 points), fair (4–5 points) and poor (less than 4 points) quality [39].

### 2.4. Statistical Analysis

The Comprehensive Meta-Analysis Software version 2.2 was employed to meta-analyze the extracted data. For each outcome, we used mean and standard deviations (SD) at baseline and post-intervention, along with the number of participants per group. If one study included two control groups, we halved the number of participants in the mindful exercise group with the two control groups, while mean and SD remained unchanged. We used random-effects model to calculate the pooled ES (standardized mean difference, SMD) to determine the magnitude of effect for mindful exercise intervention on two outcomes (pain and disability). Notably, we did not evaluate depression as an outcome variable, due to fewer than four studies evaluating this outcome [40]. Three levels of ES were adopted: small (0.2–0.49), moderate (0.50–0.79), and large (≥0.8) [40]. *I*^2^ test was used to determine heterogeneity across included studies: *I*^2^ < 25% (low), *I*^2^ < 50% (moderate), and *I*^2^ > 75% (high), respectively [40]. Furthermore, we performed sub-group analyses for categorical variables and meta-regression for continuous variables. The categorical variables included: (1) types of control condition (mindful exercise versus active control or non-active control), mindful exercise (Tai Chi, Yoga, and Qigong), and instrument; (2) use of allocation concealment. The continuous variables included mean age and total time spent over the entire intervention course (minutes). Finally, publication bias for each outcome was evaluated using the Egger’s test and the visually-produced Funnel plot [40]. Subsequently, we removed studies that caused asymmetry.

## 3. Results

### 3.1. Search Results

Figure 1 describes the detailed search process of our meta-analysis. A total of 2049 potential studies were searched and 42 full-text publications were screened for further evaluation. After eliminating the irrelevant studies (*n* = 25), seventeen studies [41,42,43,44,45,46,47,48,49,50,51,52,53,54,55,56,57] were identified for data extraction and quality assessment.

### 3.2. Characteristics of Included Studies

Table 1 depicts the characteristics of the included studies, such as the sample size, age, intervention and control group details, and outcome measures. Seventeen studies [41,42,43,44,45,46,47,48,49,50,51,52,53,54,55,56,57] published in peer-review journals included a total of 2022 participants with CLBP. The mean age of participants ranged from 34 to 74 years. The sample size ranged from 20 to 320 per study. Intervention duration for the mindful exercise(s) lasted 1 to 24 weeks, with sessions occurring one to seven times per week (40 to 90 min per sessions). Control conditions varied greatly across the evaluated studies, including utilizing a self-care book, stretching exercise, and waitlist. Adverse events were reported in five Yoga intervention studies, including herniated discs (3.3% and 1.1%, respectively) [48,54,55], increased pain (2.6% and 14.1%, respectively) [53,54], and mild self-limited joint and back pain (7.1%) [56]. One study did not report an adverse event [57], while no adverse events were reported in the other mindful exercise intervention studies.

### 3.3. Study Quality Assessment

Study quality for each evaluated experiment is summarized in Table 2. Overall, the included studies demonstrated good quality (6–8 points). Notably, no studies implemented subject blinding or therapist blinding, and only one study [56] adopted assessor blinding. Concealed allocation was conducted in 40% of the studies, and four studies did not use intention-to-treat analysis [48,49,50,55].

### 3.4. Meta-Analysis of Outcome Measured

#### 3.4.1. Pain Intensity

**Figure 2:**
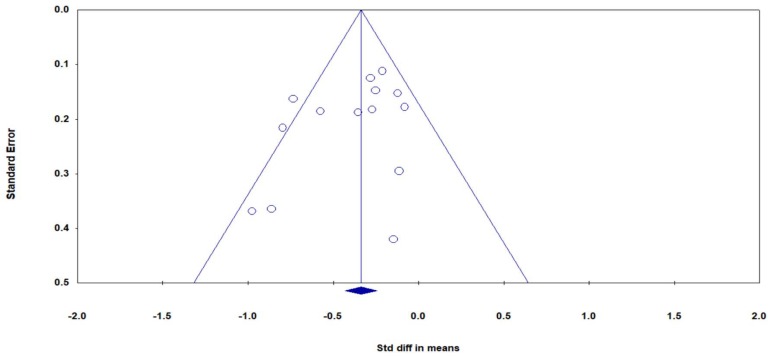
Funnel plot of publication bias for pain intensity.

**Figure 3:**
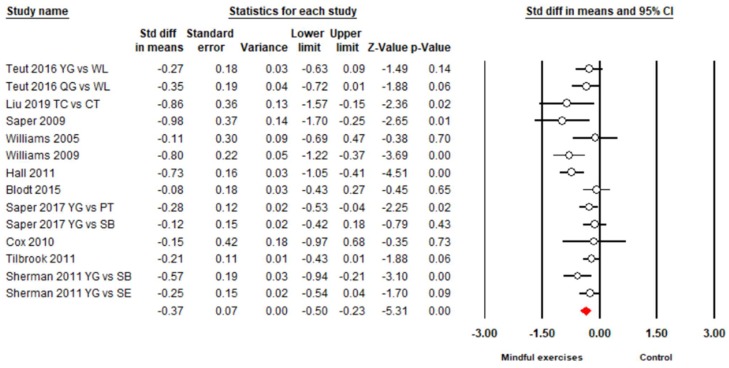
Effects of mindful exercises on pain intensity (YG = Yoga, WL = waitlist, TC = Tai Chi, CT = core training, QG = Qigong; PT = physical therapy, SB = self-care book; SE = stretching exercise). The red symbol represents the overall effect size in favor of mindful exercises.

There were 15 studies (18 pairs of intervention vs. control comparisons since three studies [43,54,56] included two control conditions) on pain intensity, measured by three different self-reported scales (Visual Analog Scale (VAS), Numeric Rating Scale (NRS), and Aberdeen Back Pain Scale (ABPS)). Based on the asymmetrical Funnel plot and the Egger’s Regression test (Egger’s regression intercept = −3.78, *p* < 0.01), we removed four comparisons [44,45,55,57] and the remaining studies showed a symmetrical Funnel plot (Figure 2) with Eggers test intercept = −1.54, *p* = 0.16. For the meta-analysis of 11 studies (14 comparisons), compared with the control groups, a significant benefit on reducing pain intensity was observed in favor of mindful exercises (*SMD* = −0.37, 95% CI −0.5 to −0.23, *p* < 0.001, *I*^2^ = 45.9%; Figure 3). Furthermore, we performed sub-group analyses and meta-regression for categorical variables (control type, type of mindful exercise, type of instrument, and use of allocation concealment) and continuous variables (mean age and total time). We observed significantly different effects on pain intensity across different types of mindful exercise (*Q* = 8.46, *p* = 0.01), with Tai Chi (*SMD* = −0.75, 95% CI −1.05 to −0.46, *p* < 0.001) and Yoga (*SMD* = −0.33, 95% CI −0.47 to −0.19, *p* < 0.001) showing significantly decreased pain intensity, but Qigong exercise did not demonstrate such an effect (*SMD* = −0.21, 95% CI −0.48 to 0.06, *p* = 0.12) (Table 3). 

#### 3.4.2. Back-Specific Disability

**Figure 4:**
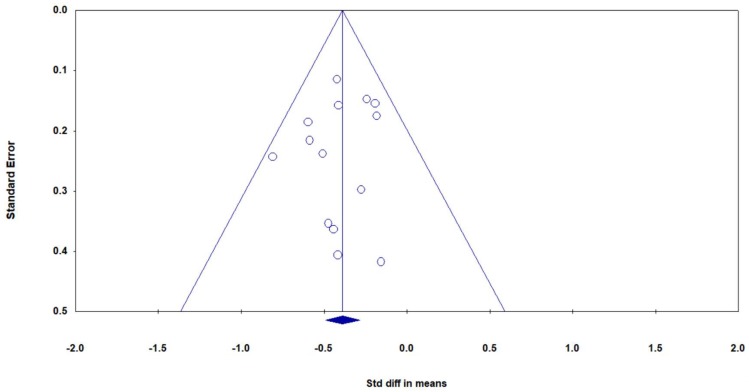
Funnel plot of publication bias for disability.

**Figure 5:**
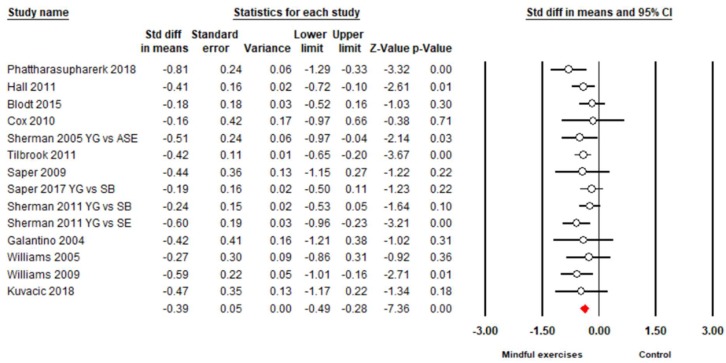
The effect of mindful exercises on disability (YG = Yoga, ASE = Aerobic and strength exercise, SB = self-care book, SE = stretching exercise).The red symbol below represents the overall effect size in favor of mindful exercises.

Overall, there were 14 studies, including 17 pairs of mindful exercises vs. control comparisons (because three studies [47,54,56] included two control conditions, respectively), with disability measured by two different types of instruments (Roland–Morris Disability Questionnaire (RMDQ) and Oswestry Disability Index (ODI)). Based on the asymmetrical Funnel plot, we removed three outlying studies [47,49,51] and the remaining studies showed a symmetrical Funnel plot (Figure 4) with Eggers test intercept = –0.42, *p* = 0.53. For the meta-analysis in 12 studies (14 pairs of mindful exercises vs. control comparisons), compared with the control groups, the aggregated result showed a significant benefit in favor of mindful exercises on reducing disability (*SMD* = −0.39, 95% CI −0.49 to −0.28, *p* < 0.001, *I*^2^ = 0%; Figure 5). We performed sub-group analyses and meta-regression for categorical variables (control type, type of mindful exercise, type of instrument, and use of allocation concealment) and continuous variables (mean age and total time) (Table 3). No significant differences were observed.

## 4. Discussion

Mindful exercises are increasingly accepted by clinicians worldwide as an alternative therapy for chronic disease symptomatic management. To the best of our knowledge, this is the first systematic review to comprehensively evaluate the existing literature regarding the safety and pain- and disease-specific effects of three commonly practiced mindful exercises (Tai Chi, Qigong, and Yoga) among adults with CLBP. Our findings indicated that mindful exercises may be effective in reducing pain intensity and disability among CLBP patients. More importantly, the beneficial effects of mindful exercises were observed comparing to both non-active and active controls. Notably, several Yoga interventions induced varied adverse events (e.g., injury).

### 4.1. Pain Intensity

Overall, mindful exercises may be effective in reducing pain intensity level, with a small intervention effect (*SMD* = −0.37). However, we observed non-significant effects on this outcome in five comparisons [42,43,48,51,52] and marginally significant effects in three comparisons [43,47,53]. Such results may be attributed to inadequacy of weekly instructor-led training time (75 to 90 min) [42,43,47,53], relatively small sample size [48,51,52] (20 to 60 participants), and/or direct comparison to active controls (strengthening or stretching exercise) [42,47]. When compared with an active control alone, mindful exercises showed significantly reduced pain intensity (*SMD* = −0.40, *p* < 0.001). This suggests that mindful exercise may be more beneficial for pain management than conventional exercise (strengthening and/or stretching exercise) and guideline-endorsed (core training or physical therapy) programs. Furthermore, results from the sub-group analyses indicated that, when compared to Yoga and Qigong, Tai Chi appeared to have a superior effect on pain relief. Such positive intervention effects reached a moderate level (*SMD* = −0.75). Tai Chi emphasizes neutral spine or standing with upright posture during performance, providing an opportunity to strengthen core muscles (similar to a guideline-endorsed core training program) to reduce pain intensity. Additionally, a previous RCT by Hall [58] indicated that Tai Chi can reduce pain-catastrophizing, which partially mediates the effect of Tai Chi on pain intensity among adults with CLBP. Conversely, adverse events (increased pain, reduced range of motion at joints, and/or herniated disc) were reported in several Yoga intervention studies but not in Tai Chi studies. This is likely due to the Yoga routine, which involves movements of bending forward and backwards at the low back, which may initiate or exacerbate pain intensity. Taken together, Tai Chi may be a more suitable mindful exercise in rehabilitation programs for CLBP rather than Yoga.

### 4.2. Back-Specific Disability

In this meta-analysis, we observed a small overall positive effect (SMD = −0.39) of mindful exercise on disability. Of the 12 studies (including 14 comparisons), six comparisons (Qigong vs. waitlist, Tai Chi vs. waitlist, Yoga vs. aerobic plus strength exercises, Yoga vs. Waitlist, Yoga vs. self-care book, Yoga vs. stretching exercise, and Yoga vs. waitlist) [41,44,47,50,53,54] showed significant effects on CLBP-specific disability, whereas the other eight [42,46,48,51,52,54,56,57] demonstrated positive effects. Throughout the 12-week intervention period, weekly instructor-based training length ranged from 75 to 90 min in Qigong [42] and Yoga [54,56], which may not be sufficient to achieve significant reductions in disability risk. Notably, Neiyanggong, as one type of Qigong exercise, is not as popular as Baduanjin and Wuqinxi Qigong. Thus, it presumably takes beginners much longer to understand the principle and movement concepts, particularly during the initial stage of motor learning (cognitive stage) [59]. A 90-min session per week during a 12-week Neiyanggong intervention may not be sufficient to maximize the potential benefits of this modality of exercise. Likewise, movements in Yoga routine are relatively complex and require a certified instructor, and self-practice at home may lead to incorrect movement patterns, which may have contributed to the deterioration in disability or caused the observed adverse events (increased pain, herniated disc, and/or reduced range of motion at joints) reported in the five Yoga intervention studies [48,53,54,55,56]. Second, three studies included relatively small sample sizes of 20 [52], 30 [51] and 60 participants [48], which may have affected the power of detecting significant differences on disability risk.

### 4.3. Strengths and Limitations for Future Research

Strengths of this systematic review are as follows: (1) we provide a comprehensive review regarding the effectiveness of mindful exercises on CLBP disease-specific symptoms; (2) we were the first to include three popular mindful exercises; (3) we compared mindful exercises with active controls (conventional exercises and guideline-endorsed physical therapy); and (4) we evaluated the safety of mindful exercises in adults with CLBP [60,61]. Several limitations should be considered: (1) this review only included English-language studies, which possibly excluded Chinese-language journals that may be more likely to publish Tai Chi and Qigong studies; (2) we limited our meta-analysis to pain intensity and disability. We were not able to meta-analyze data on depressive symptomology (and other related outcomes) due to fewer than four studies reporting data on this outcome. Thus, future studies should include psychological outcome measures; (3) blinding of assessors was only used in one study (blinding of instructor and participants are, however, unrealistic), and it remains unclear whether greater expectations were associated with reduced pain intensity and disability in the mindful exercise groups; (4) some studies did not use “intention to treat analysis” and “allocation concealment”, which possibly overestimated the pooled effect size; (5) none of studies used follow-up assessments, so it is difficult to determine how long the beneficial effects of mindful exercise interventions lasted in adults with CLBP; (6) previous studies suggest that different brain mechanisms are associated with different mindful exercises, thus, future studies should comparatively investigate different mind-body exercises as well as their underlying mechanisms [62,63].

## 5. Conclusions

Findings of our systematic review suggest that mindful exercises (Yoga, Tai Chi, and Qigong) may be beneficial for CLBP symptomatic management, irrespective of non-control comparison or active control comparison (conventional exercises, core training, and physical therapy programs). The potential of Tai Chi as a routine non-pharmacological approach for CLBP needs to be rigorously evaluated in future studies. Importantly, training in these mindful exercises should be implemented with certified instructors, to ensure quality of movement and injury prevention. Before definitive conclusions can be drawn, future work is needed that employs more robust study designs and implements long-term follow-up assessments.

## Figures and Tables

**Table 1:** Characteristics of randomized controlled trials in the meta-analysis.

Study	Participants		Intervention Protocol				Outcome Measured	Safety
	Sample Size	MA (years)	Mindful Exercise (Qualified Instructor)	Control	DR (wk)	Control Type	Pain and Disability	Adverse Events
Hall et al., (2011) [41]	160 CLBP TC = 80; C = 80	44	2 × 40 min/wk, TC	Wait-list	10	Passive	Pain intensity (NRS), disability (RMDQ)	No adverse event
Blödt et al., (2015) [42]	127 CLBP QG = 64; C = 63	47	1 × 90 min/wk, QG	1 × 60 min/wk Strengthening	12	Active	Pain intensity (VAS), disability (RMDQ)	No adverse event
Teut et al., (2016) [43]	176 CLBP QG = 58; YG = 61 C = 57	73	1 × 90 min/wk, QG; 2 × 45 min/wk, YG	Waitlist	12	Passive	Pain intensity (VAS)	No adverse event
Phattharasupharerk et al., (2018) [44]	72 CLBP QG = 36; C = 36	35	1 × 60 min/wk plus daily practice, YG	Waitlist	6	Passive	Pain intensity (VAS), disability (RMDQ)	No adverse event
Liu et al., (2019) [45]	43 CLBP TC = 15; C1 = 15; C2 = 13	74	3 × 60 min/wk, TC	C1: Core training C2: No intervention	12	C1: Active C2: Passive	Pain intensity (VAS)	No adverse event
Galantino et al., (2004) [46]	22 CLBP YG = 11; C = 11	30–65	2 × 60 min/wk plus 7 × 60 min/wk (home), YG	No treatment	6	Passive	disability (ODI) Depression (BDI)	No adverse event
Sherman et al., (2005) [47]	101 CLBP YG = 36; C1 = 35; C2 = 30	44	1 × 75 min/wk plus daily practice (home), YG	C1: 1 × 75min/wk + Daily practice, aerobic exercises and strength exercise C2: Self-care book	12	C1: Active C2: Passive	disability (RMDQ)	No adverse event
Williams et al., (2005) [48]	60 CLBP YG = 30; C = 30	48	1 × 90 min/wk plus 5 × 30 min/wk (home), YG	Newsletters on back pain	16	Passive	Pain intensity (VAS), disability (ODI)	1 participant diagnosed with a herniated disc in YG
Tekur et al., (2008) [49]	80 CLBP YG = 40; C = 40	48	7 × 120 min/wk, YG	Daily physical movements + education	1	Active	disability (ODI)	No adverse event
Williams et al., (2009) [50]	90 CLBP YG = 43; C = 47	48	2 × 90 min/wk plus 7 × 30 min/wk (home), YG	Waitlist	24	Passive	Pain intensity (VAS), disability (ODI)	No adverse event
Saper et al., (2009) [51]	30 CLBP YG = 15; C = 15	44	1 × 75 min/wk plus 7 × 30 min/wk (home), YG	Self-care book	12	Passive	Pain intensity (VAS), disability (RMDQ)	No adverse event
Cox et al., (2010) [52]	20 CLBP YG = 10; C = 10	45	1 × 75 min/wk plus home practice, YG	Self-care book	12	Passive	Pain intensity (ABPS), disability (RMDQ)	No adverse event
Tilbrook et al., (2011) [53]	313 CLBP YG = 156; C = 157	46	1 × 75min/wk plus 7 × 30 min/wk (home), YG	Self-care book	12	Passive	Pain intensity (ABPS), disability (RMDQ)	8 participants (increased pain) in YG
Sherman et al., (2011) [54]	228 CLBP YG = 92; C1 = 9 1; C2 = 45	48	1 × 75 min/wk plus 6 × 20 min/wk (home),YG	C1: 1 × 75min/wk + 20 min/wk (home) Stretching exercise C2: Self-care book	12	C1: Active C2: Passive	Pain intensity (NRS) disability (RMDQ)	13 participants (increased pain) and 1 herniated disc in yoga
Nambi et al., (2014) [55]	60 CLBP YG = 30; C = 30	44	1 × 60 min/wk plus 5 × 30 min/wk (home), YG	35days/wk, Exercise (strengthening and stretching)	4	Active	Pain intensity (VAS)	1 herniated disc in YG
Saper et al., (2017) [56]	320 CLBP YG = 127; C1 = 129; C2 = 64	46	1 × 75 min/wk plus 7 × 30 min/wk (home), YG	C1: 1 x 60min/wk, PT (stabilization and aerobic exercise) C2: Self-care book	12	C1: Active C2: Passive	Pain intensity (NRS) disability (RMDQ)	9 and 14 participants (mild self-limited joint and back pain) in YG and PT, respectively
Kuvačić et al., (2018) [57]	30 CLBP YG = 15; C = 15	34	2 × 75 min/wk, YG	Pamphlet program	8	Passive	Pain intensity (NRS), disability (ODI), depression (SDS)	Not reported

Note: TC = Tai Chi; YG = Yoga; QG = Qigong; PT = Physical therapy; = control group; MA = mean age; wk = week; DR = duration; CLBP = Chronic lower back pain; VAS = Visual Analog Scale; NRS = Numeric Rating Scale; ABPS = Aberdeen Back Pain Scale; ODI = Oswestry Disability Index; RMDQ = Roland–Morris Disability Questionnaire; Self-care book refers to reading *The Back Pain Book*, which emphasizing self-care management strategies for low back pain such as the causes of back pain and advice on exercising, appropriate lifestyle modification, and guidelines for managing flare-up; Pamphlet program refers to knowledge about vertebral spine and its biomechanical aspects; BDI = Beck depression inventory; SDS = Zung self-rating depression scale.

**Table 2:** Methodological quality of the included studies (PEDro assessment).

Study	Score	Methodological Quality	PEDro Item Number										
			1	2	3	4	5	6	7	8	9	10	11
Hall et al., 2011 [41]	8	Good	✔	✔	✔	✔				✔	✔	✔	✔
Blödt et al., 2015 [42]	8	Good	✔	✔	✔	✔				✔	✔	✔	✔
Teut et al., 2016 [43]	8	Good	✔	✔	✔	✔				✔	✔	✔	✔
Phattharasupharerk et al., 2018 [44]	7	Good	✔	✔		✔				✔	✔	✔	✔
Liu et al., 2019 [45]	7	Good	✔	✔		✔				✔	✔	✔	✔
Galantino et al., 2004 [46]	7	Good	✔	✔		✔				✔	✔	✔	✔
Sherman et al., 2005 [47]	8	Good	✔	✔	✔	✔				✔	✔	✔	✔
Williams et al., 2005 [48]	6	Good	✔	✔		✔				✔		✔	✔
Tekur et al., 2008 [49]	7	Good	✔	✔	✔	✔				✔		✔	✔
Williams et al., 2009 [50]	6	Good	✔	✔		✔				✔		✔	✔
Saper et al., 2009 [51]	8	Good	✔	✔	✔	✔				✔	✔	✔	✔
Cox et al., 2010 [52]	8	Good	✔	✔	✔	✔				✔	✔	✔	✔
Tilbrook et al., 2011 [53]	8	Good	✔	✔	✔	✔				✔	✔	✔	✔
Sherman et al., 2011 [54]	8	Good	✔	✔	✔	✔				✔	✔	✔	✔
Nambi et al., 2014 [55]	6	Good	✔	✔		✔				✔		✔	✔
Saper et al., 2017 [56]	9	Excellent	✔	✔	✔	✔			✔	✔	✔	✔	✔
Kuvačić et al., 2018 [57]	7	Good	✔	✔		✔				✔	✔	✔	✔
Studies were classified as having excellent (9–10), good (6–8), fair (4–5) or poor (<4)													

Scale of item score: ✔, present. The PEDro scale criteria are (1) eligibility criteria; (2) random allocation; (3) concealed allocation; (4) similarity at baseline on key measures; (5) subject blinding; (6) therapist blinding; (7) assessor blinding; (8) more than 85% follow-up of at least one key outcome; (9) intention-to-treat analysis; (10) between-group statistical comparison for at least one key outcome; and (11) point estimates and measures of variability provided for at least one key outcome.

**Table 3:** The effect of mind-body exercise in moderator analysis.

Categorical Moderator	Outcome	Covariates	No. of Studies/Comparisons	SMD	95% Confidence Interval	*I*^2^%	Test for Between-Group Hoterogeneity		
							*Q*-Value	df(*Q)*	*p*-Value
Control Type	Pain intensity	Active	7	−0.40	-0.48 to -0.20	53.2 %	0.08	1	0.78
		Passive	7	-0.35	−0.46 to −0.21	46.5%			
	Disability	Active	4	−0.28	−0.47 to −0.09	0%	1.62	1	0.20
		Passive	10	−0.43	−0.55 to −0.31	0%			
Mindful Type	Pain intensity	Yoga	10	−0.33	−0.47 to −0.19	33.7%	8.46	2	0.01*
		TC	2	−0.75	−1.05 to −0.46	0%			
		Qigong	2	−0.21	−0.48 to 0.06	10.0%			
	Disability	Yoga	11	−0.38	−0.50 to −0.26	0%	0.16	2	0.92
		TC	1	−0.41	−0.72 to −0.10	0%			
		Qigong	2	−0.47	−1.09 to 0.14	77.2%			
Instruments	Pain intensity	ABPS	2	−0.21	−0.42 to 0.01	0%	2.1	2	0.35
		VAS	7	−0.43	−0.68 to −0.18	50.5%			
		NRS	5	−0.38	−0.59 to −0.17	60.1%			
	Disability	RMDQ	10	−0.38	−0.49 to −0.27	0%	0.36	1	0.55
		ODI	4	−0.47	−0.76 to −0.18	0%			
Allocation Concealment	Pain intensity	Yes	11	−0.33	−0.46 to −0.19	39.5%	1.19	1	0.28
		No	3	−0.59	1.05 to −0.13	50.9%			
	Disability	Yes	9	−0.35	−0.46 to −0.24	0%	2.27	1	0.13
		No	5	−0.56	−0.80 to −0.31	0%			
**Continuous moderator**	**Outcome**	**No. of studies/comparisons**	***β***	**95% Confidence Interval**		***Q*-value**		**df(*Q*)**	***p*-value**
Age	Pain intensity	14	−0.00108	−0.01080 to 0.00865		0.05		1	0.83
	Disability	14	0.02454	−0.00706 to 0.05614		2.32		1	0.13
Total Time	Pain intensity	14	0.00002	−0.00007 to 0.00012		0.22		1	0.64
	Disability	14	−0.00002	−0.00012 to 0.00009		0.10		1	0.75

VAS = Visual Analog Scale; RMDQ = Roland-Morris Disability Questionnaire; SMD = Standardized Mean Difference; TC = Tai Chi; * *p* < 0.01.
